# Bovine serum albumin-functionalized graphene-decorated strontium as a potent complex nanoparticle for bone tissue engineering

**DOI:** 10.1038/s41598-022-16568-7

**Published:** 2022-07-19

**Authors:** Hossein Akbari, Esfandyar Askari, Seyed Morteza Naghib, Zeinab Salehi

**Affiliations:** 1 School of Chemical Engineering, College of Engineering, University of Tehran, Tehran, Iran; 2 Biomaterials and Tissue Engineering Research Group, Department of Interdisciplinary Technologies, Breast Cancer Research Center, Motamed Cancer Institute, ACECR, Tehran, Iran; 3 Nanotechnology Department, School of Advanced Technologies, Iran University of Science and Technology (IUST), P.O. Box 16846-13114, Tehran, Iran

**Keywords:** Implants, Biomaterials, Tissues

## Abstract

Graphene and its family have a great potential in tissue engineering because of their super mechanical properties, electrical conductivity and antibacterial properties. Considering other properties of graphene such as high surface area and ready-to-use functionalization according to the high oxygen-containing groups in graphene oxide family, some needs could be addressed in bone tissue engineering. Herein, we synthesized and decorated strontium nanoparticles (SrNPs) during the reduction process of graphene oxide using green and novel method. Without using hydrazine or chemical linkers, strontium NPs were synthesized and decorated on the surface of rGO simultaneously using BSA. The results of the UV–Vis, FTIR and Raman spectroscopy demonstrated that BSA could successfully reduce graphene oxide and decorated SrNPs on the surface of rGO. FESEM and TEM exhibited that in situ synthesized SrNPs had 25–30 nm diameter. Interestingly, cell viability for MC3T3-E1 cells treated with SrNPs-rGO, were significantly higher than BSA-rGO and GO in constant concentration. Furthermore, we investigated the alkaline phosphatase activity (ALP) of these nanosheets that the results demonstrated Sr-BSA-rGO enhanced ALP activity more than GO and BSA-rGO. Remarkably, the relative expression of RUNX 2 and Col1 genes of MC3T3-E1 cells was boosted when treated with Sr-BSA-rGO nanosheets. This study revealed that using proteins and other biomolecules as green and facile agent for decoration of smart nanoparticles on the surface of nanosheets, would be promising and assist researcher to replace the harsh and toxic hydrazine like materials with bio-friendly method. These results demonstrated that Sr-BSA-rGO had the excellent capability for regenerating bone tissue and could be used as an osteogenesis booster in implants.

## Introduction

Bone is one of the most critical tissue in the body, because it serves as a foundation for mechanical support, organ protection and skeletal continuity. Damage to the bone structural integrity may occur for various causes, including trauma, surgery, tumors and osteoporosis^1^. In most cases, bone has a significant capacity to regenerate and repair itself. However, there are certain situations in which full regeneration of bone tissue is not feasible and needs further stimulation^2^. Biomaterials are a viable alternative to bone transplants in bone tissue engineering^3,4^. Synthetic hydroxyl apatite, tri-calcium phosphate, other bioceramics, polymeric scaffolds and metallic implants are examples of biomaterials widely utilized in bone and hard tissue engineering^4^. Nanotechnology advancements have transformed nanomedical research in clinical science, resulting in novel nanodevices and nanosystems that relied on the design and perfect integration of functional nanomaterials. The graphene family derivatives have received much interest in biomedical applications among many synthetic nanobiomaterials^5,6^. A hydrophilic version of graphene sheet with hybridized sp^2^ carbon atoms known as graphene oxide (GO), has emerged as a promising biomedical application^7^. The reduced form of graphene oxide, which had minimal toxicity, biocompatibility and reaction sites may be utilized to stimulate cellular activities and increased the osteogenic capacity of bone tissue^8–10^. The orthopaedic therapeutic properties of strontium (Sr) have generated interest in this context^11^. Human hard tissues may accumulate Sr which can displace calcium from the apatite phase of bone mineral. Sr is also associated with an increase in bone compressive strength, whereas its shortage is associated with negative consequences in hard tissues. In vitro and in vivo studies have demonstrated that Sr ions stimulated bone formation and inhibited bone resorption which made them a potential agent for the treatment of osteoporosis^12^. The properties of Sr have made it a popular ingredient in bioactive glasses and biocermaics due to its advantages^13^. Sr has been used in bone remodelling applications because of its structural and physico-chemical similarity to calcium ions (Ca^2+^). For instance, the incorporation of Sr into calcium phosphate and titanium implants has been investigated to enhance bone-forming properties of these materials^14^. Due to the large surface area of graphene nanosheets, recently, researchers have synthesized SrNPs on the surface of graphene by co-reduction of GO and Sr^15^. Hydrazine as a reducing agent, was used to reduce and despite SrNPs on the surface of rGO reported by Kumar et al.^16^. Recently, Qi et al. synthesized Sr-decorated rGO nanosheets using hydrazine incorporated into the Poly(L-lactide) (PLLA) for fabrication of 3D scaffold. Osteogenesis induction in Sr-rGO, was exceled to rGO and pure PLLA scaffolds^15^. However, these studies demonstrated high capability of the Sr-rGO nanosheets for bone tissue engineering, because enhancing graphene strength and Sr osteogenesis properties using hydrazine in the synthesis process, had major concerns. First, water solubility of rGO nanosheets have decreased significantly that caused difficulties in fabrication process of graphene-based composite. Second, hydrazine was toxic reagent that affected user-health during the experiment^17^. Many efforts have been made to introduce green and safe reducer to overcome this limitation of hydrazine. For example, protein like bovine serum albumin (BSA) and ascorbic acid are the safe reducers for GO^3,18,19^.

Proteins are complex biopolymers with hydrophobic and hydrophilic segments, that may serve as an adhesive for solid surfaces^20^. BSA is a spherical shape protein which has 583 amino acid residues^21^. In the structure of BSA, there is tyrosine residues which distinguishes it as a distinct reducing agent^20^. Hydrophobic sections of BSA may be adsorbed to hydrophobic surface area, whereas hydrophilic parts of BSA could be interacted with water functional groups in the presence of oxygen^22^.

**Figure 1:**
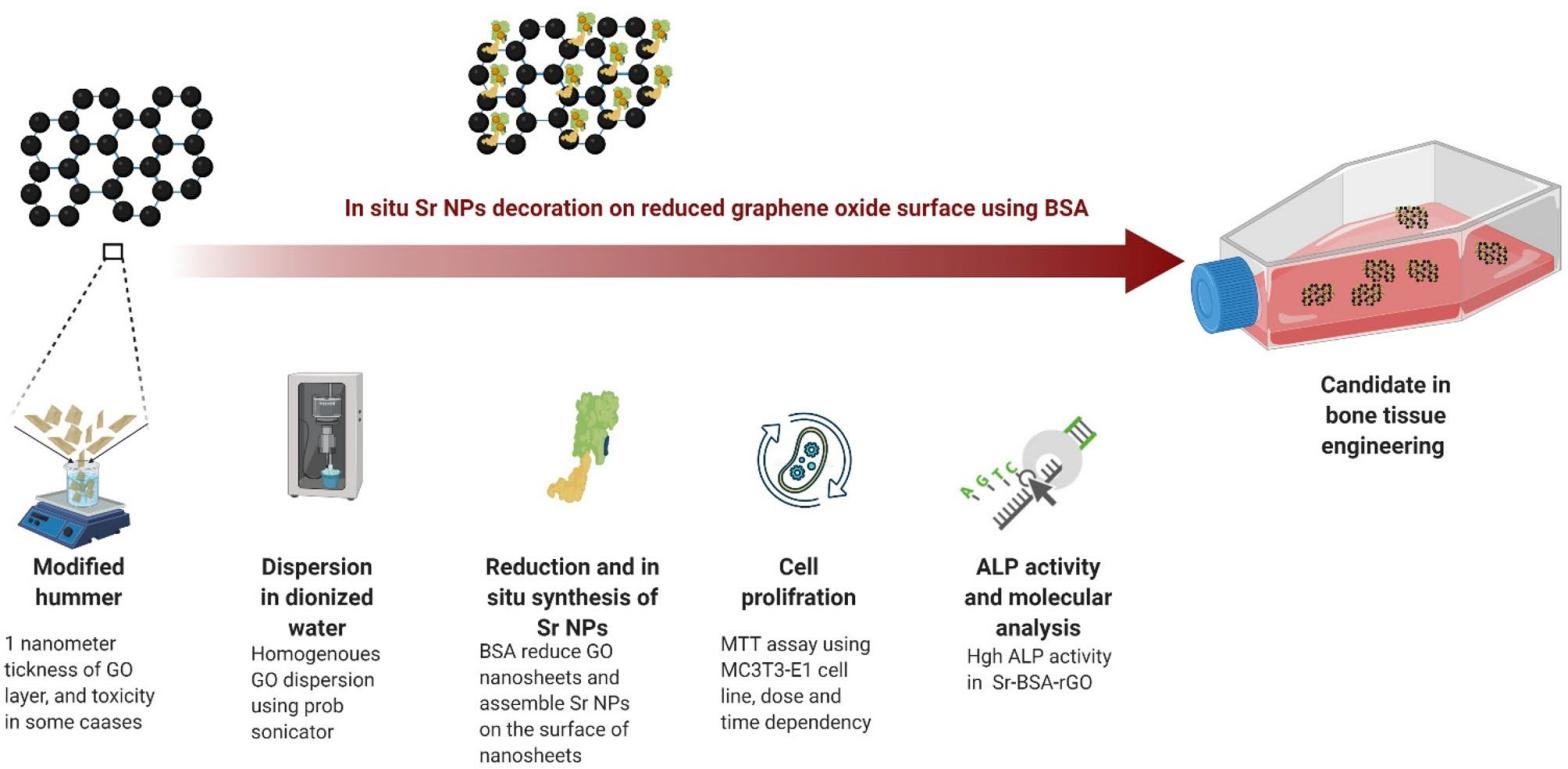
Sr-BSA-rGO nanosheets in bone tissue engineering. This illustration demonstrates the synthesis and application process from chemistry lab to molecular genetic lab, that was employed in this study.

In this study, graphene-decorated Sr nanosheets were synthesized by co-reduction of GO and strontium nitrate in the presence of BSA, named Sr-BSA-rGO. Synthesis process is illustrated in Fig. 1. In Sr-BSA-rGO, SrNPs have been located on the surfaces of rGO nanosheets, and in close collaboration with BSA that acts as steric hindrance to block re-stacking of rGO nanosheets after reduction. Then, the presence of SrNPs on the surface of rGO, was studied using different methods. In following, osteoblast cell line was utilized for investigating the effect of BSA-rGO-decorated Sr on the proliferation. Alkaline phosphatase (ALP) activity that is important factor in bone tissue engineering, was also conducted. Finally, COL1 and RUNX2 genes expression were revealed using Real-Time PCR.

## Materials and methods

### Materials and equipment

Graphite, KMnO_4_, Sulfuric acid (H_2_SO_4_) were purchased from Merck. BSA, Strontium nitrate (SrNO_3_) were purchased from Sigma. All reagents were used without purification.

### Synthesis of graphene oxide

In line with the modified Hummer's technique, graphene oxide powder was produced by the chemical oxidation of black graphite in a laboratory setting. In this method, 1 g graphite was exposed to 23.3 ml H_2_SO_4_ in an ice bath, which graphite was inserted between the acid medium. Oxidation agent replaced the acid insertion to produce the pristine graphite oxide. In order to generate pure graphite oxide (PGO), KMnO_4_ was used as an oxidant and was added progressively from 5 to 15 °C^23^. Purification of impurities was carried out in order to convert graphite to graphite oxide (GO). PGO hydrolysis was used to eliminate the remaining sulphate contaminant. To eliminate the covalent sulfate of PGO, 150 ml distilled water was added and temperature was arisen to 90 °C in the stirring condition within 30 min, and the system diluted via 500 ml distilled water again. For instance, hydrogen peroxide (H_2_O_2_) was added dropwise instantly, to subtract unreacted KMnO_4_ that changed the colour to dark brown^24^. The graphite oxide solution was filtered and washed with diluted HCl and ethanol in the centrifuge at 8000 rpm, and dried at room temperature. An ultrasonic process was conducted to exfoliate the GO nanosheets. The graphite oxide powder was dispersed in the deionized water (10 mg/ml), and subsequently was sonicated at power 30 w for 3 h (note that the temperature of the solution should be at 4 °C using ice-bath).

### Decoration of SrNPs on the surface of GO nanosheets

We used BSA as a green reducing agent and a bio glue, to bind Sr to the GO surface. Initially, 0.5 g of GO was dispersed in deionized water (200 ml) using ultrasound, to exfoliate the GO layer in water. 2.5 g of BSA was added to the exfoliated GO solution under stirring conditions. To make a reaction between BSA and GO, the pH of the solution increased to 11.5 by adding 1 M NaOH dropwise. The GO-BSA mixture was blended for 24 h, and the soluble impurities were separated by ethanol centrifugation. For the production of Sr-BSA-rGO, the strontium nitrate was combined with GO in different concentrations (5, 10, 20 wt% of GO). Briefly, BSA-rGO was dispersed and sonicated in deionized water for 1 h (power: 30 W). Then, strontium nitrate was added to the rGO solution and stirred for 2 h to obtain a homogeneous mixture. The mixture was then washed with ethanol.

### Characterization

This study used X-ray diffraction analysis on the produced powders using a PHILIPS- PW1730 diffractometer with Cu-K radiation, to determine the phase and crystallinity. The microstructural characteristics of the generated nanosheets were investigated using a field emission scanning electron microscope (FESEM, Hitachi S-3400 N, voltage of 20 kV) and a transition electron microscope (TEM, Philips, CM300). Raman spectroscopy was conducted using TakRam N1-541, Teksan Co, Iran. FTIR spectrums were recorded using TENSOR 27 Brucker.

### Cell culture

We utilized MC3T3-E1 cell line taken from the National Cell Bank of Iran (NCBI) at Pasteur Institute of Iran (IPI). The cells were allowed to grow in Dulbecco's Modified Eagle's Medium (DMEM) in neutral pH (7.2–7.4), which supplemented with 10% (v/v) heat-inactivated (50 °C, 30 min) Fetal Bovine Serum (FBS, 10% v/v), 2 mM L-glutamine, 100 units/mL of penicillin and 100 mg/mL of streptomycin at 37 °C and 5% CO_2_ in a humidified incubator. Then, cells were trypsinized (0.025% trypsin, 0.02% EDTA) after they were grown until 70–80% confluent. Prior to treatments, cells were allowed overnight to reattach to the bottom of 96 wells cell culture plate.

### MTT assay for MC3T3-E1 cells treated by the nanosheets

Effect of Sr-BSA-rGO, BSA-rGO (named as rGO in graphs) and GO treatments on the MC3T3-E1 cell viability, was determined using the 3-(4,5-dimethylthiazol-2-yl)-2,5-diphenyltetrazolium bromide (MTT) assay at each desired treatment time. Working solutions (1, 10, 50 and 100 μg/ml) of Sr-BSA-rGO nanosheets were prepared in DMEM medium with 1 mg/ml stock solution (dispersed in DMEM medium). We also used GO and BSA-rGO nanosheets solution (concentration 100 μg/ml) to study the effect of Sr nanoparticles. After treatment, plats were incubated for 24, 72, and 120 h to study the effect of different incubation times on the cell viability. In each time point, 20 µl of MTT solution (5 mg/ml) was added into each well of cell culture (media volume: 200 μl). The plate was incubated at 37 °C for 4 h. After incubation, the previous solution was slowly removed and followed by 100 μl of DMSO solution added to each well. The cell culture plates were returned to the incubator for 1 h. The absorbance was measured at 570 nm using BioTek plate reader.

### Alkaline phosphatase activity measurement

ALP is one of the most factors that should be measured in bone tissue engineering. After measuring viability of cells treated by Sr-BSA-rGO, BSA-rGO and GO nanosheets at different incubation times, we selected the highest concentration of Sr-BSA-rGO (100 μg/ml) and the constant concentration of BSA-rGO and GO nanosheets (100 μg/ml) for studying ALP activity of MC3T3-E1 cells. The cells were cultured on the 6 well plates (100 × 10^3^ cell per well) and incubated overnight to adhere on the surface of the plate. In the following, media were replaced by the working solutions of nanosheets and harvested in the incubator. ALP kit (Pars Azmun, Iran) was utilized for the experiment according to the manufacturing protocol. Absorbance of the wells was recorded using 405 nm plate reader.

### Osteogenic gene expression

The osteogenesis-related gene expression was examined to investigate the osteogenic differentiation potential of cells cultured on Sr-BSA-rGO treated samples. In brief, the cells were harvested at a density of 104 cells per well in a six-well plate and cultivated overnight. Following that, the Sr-BSA-rGO nanosheets were mixed into culture media (100 μg/ml) and cultured for 1, 3 and 5 days, and the wells were considered as control (without nanosheets). The cells were then washed three times with PBS solution before being digested with 0.25 percent trypsin. The cell RNA was extracted with the RNX reagent (Zist Idea) and reverse-transcribed to cDNA with the PrimeScript 1st strand cDNA synthesis kit (Iran). Finally, the levels of Col1 and runt-related transcription factor-2 (Runx2) were determined. Each group was examined three times. The details of the primer design used in this study, are in Table 1.

**Table 1:** Primers design details used in this study.

Name	Primer sequence	Annealing	Tm
Col1-F	GCTTCAGGGAGTGCCATCAT	55	61
COL1-R	ACTCACATTGGAGCCACTAGGAAT		60
RUNX2-F	GAGTGGACGAGGCAAGAGTT	54	60
RUNX2-R	GCTTCTGTCTGTGCCTTCTG		59

### Statistical analysis

All data was collected in triplicate, and graphs were generated using Graphpad prism 8 software. Anova two-way was used to study the significance of differences between the groups.

## Results and discussion

**Figure 2:**
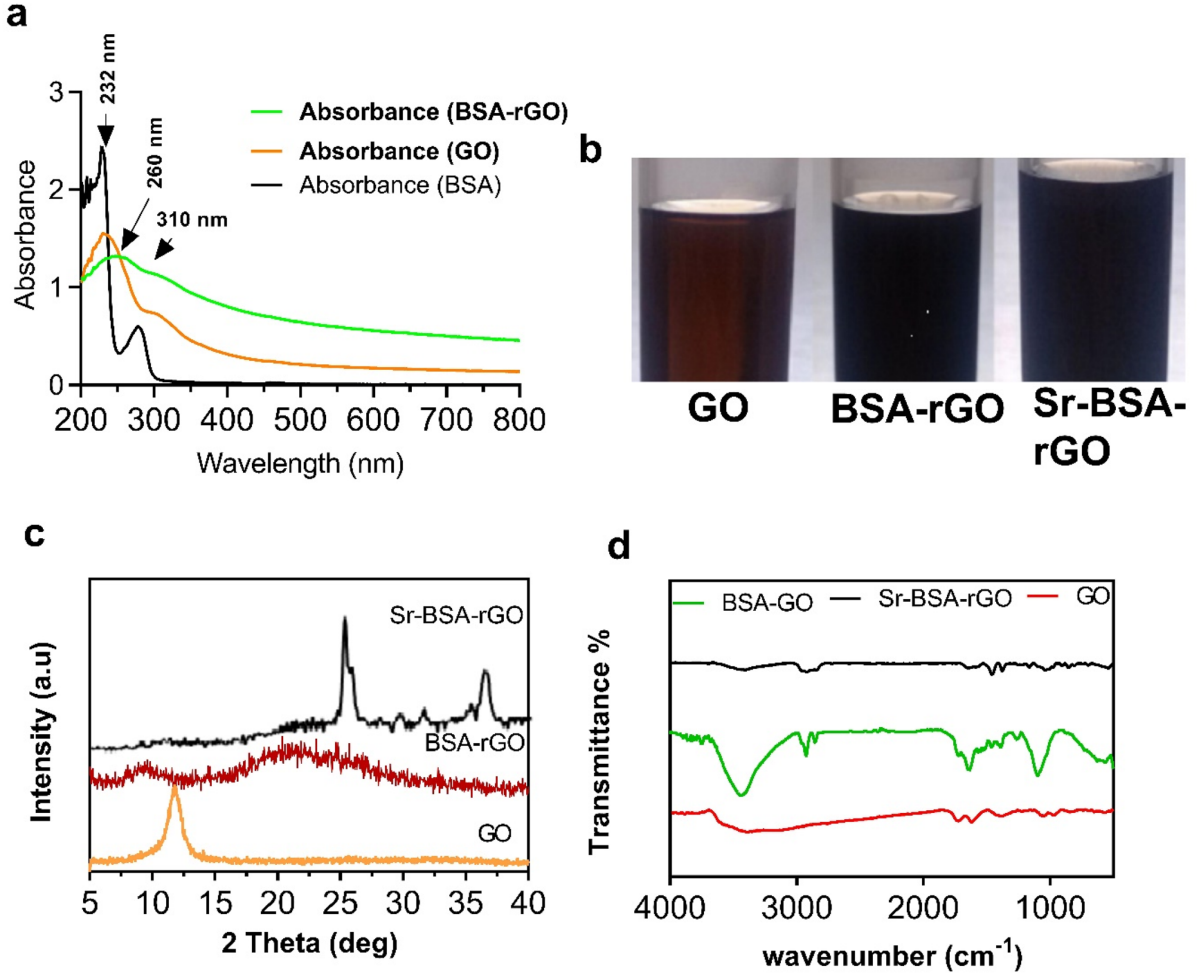
Characterization of Sr-BSA-rGO nanosheets. (**a**) The UV–Vis spectrums of BSA, GO and BSA-rGO dispersed in deionized water (concentration: 0.1 mg/ml). (**b**) Images of the synthesized GO, BSA-rGO and Sr-BSA-rGO. (**c**) and (**d**) XRD and FTIR profiles of GO, BSA-rGO and Sr-BSA-rGO nanosheets respectively.

Figure 2a shows the UV–Vis spectrums of GO, BSA-rGO and BSA. GO, a passion form of graphene families, showed a unique UV–Vis spectrum. In these spectra, the characteristic peaks of GO were 225 and 310 nm attributing to π-π and n-π interactions, respectively. The exfoliation and oxidation process of the produced graphene have been confirmed by appearing these peaks. After reducing GO with BSA, the peak at 232 nm, was shifted to 260 nm, and the intensity of 310 nm peak was significantly decreased. By administrating the BSA molecules into the GO, these peaks shifted or disappeared due to the reduction process. We found that the brownish color of GO was changed to black after reduction, and black color was stable when Sr was added to BSA-rGO mixture (Fig. 2b, images were recorded 1 week after synthesis). The XRD patterns of GO, BSA-rGO and Sr-BSA-rGO are visible in Fig. 2c. The GO corresponded pattern shows a characteristic peak at 2θ = 12.3° ascribed to the (001) spacing. According to the scherrer equation and d-spacing for the produced graphene oxide, the stacking height and the number of layers are 8.9 nm and 8, respectively. Because of the amide bond of BSA, the broad and rather strong peak positioned in the scope of 20–27°, was detected in BSA-rGO. Meanwhile, due to the homogeneous dispersion of GO inside BSA, there is no characteristic GO peak in the XRD patterns of the BSA-rGO composites, implying long-range disorder or complete exfoliation of GO in the BSA-rGO, that the result is consistent with prior research^25^. In the final XRD pattern, the characteristic peaks of metallic strontium and rGO nanosheets were observable. The peaks at 25.42° and 29.46°, were corresponded to [111] and [200] crystal planes of the cubic close-packed structure of strontium (JCPDS 89-4045), whereas the weak and broad peaks of BSA-rGO could be detected at 20–27°. The reduction in the rGO peak intensity, shows that metallic SrNPs clinging to its surface, prevented BSA-rGO nanosheets from restacking, resulting in better exfoliated Sr-BSA-rGO nanosheets than BSA-rGO nanosheets.

It has been demonstrated that growing metallic NPs on GO or rGO, causes stacked graphitic peaks to vanish or diminish, because the metallic particles impede restacking. The existence of crystalline metallic SrNPs on the well exfoliated rGO surface, was validated by XRD data, which indicated the synthesis of GO and the serum reduction of GO to rGO. The FTIR spectrums of the synthesized GO, BSA-rGO and Sr-BSA-rGO have been presented in the Fig. 2d. The characteristic GO peaks in the FTIR spectrum correspond to 3386 cm^−1^, 1728 cm^−1^ and 1615 cm^−1^ with the O–H, C=O and C=C bonds, respectively^26^. Moreover, the peaks at 1050 cm^−1^ and 1224 cm^−1^, relate to C–O stretching vibration, and also, the feature peak of 1376 cm^−1^ belongs to the deformation vibration of C–O^27^. There have been several changes to the BSA-rGO pattern compared to the GO. The peak at 630 cm^−1^, which was specific to the blending vibration of O=C–NH, was related to the bonding of the BSA deposited on GO compound. The new peak appeared at 2850 cm^−1^, was for the C–H stretching vibrations of the BSA methylene functional group^25^. In summary, for GO and BSA-rGO nanosheets, the appearance of peaks at 630 cm^−1^ and 2850 cm^−1^ in BSA-containing compounds, unlike GO nanoparticles, confirmed the formation of BSA-rGO composite. The decrease in the Sr-BSA-rGO at 630 cm^−1^ and 2850 cm^−1^, could be evidenced and the positively charged SrNPs were strongly decorated on the surface of rGO nanosheets. In other hand, we observed the reduction in the hydroxyl groups by introducing strontium nitrate. According to the previous reports, hydroxyl groups could interact with Sr^2+^ ions because of electronegativity and after Sr decoration, the intensity of hydroxyl groups in FTIR spectra, would be reduced^28^.

**Figure 3:**
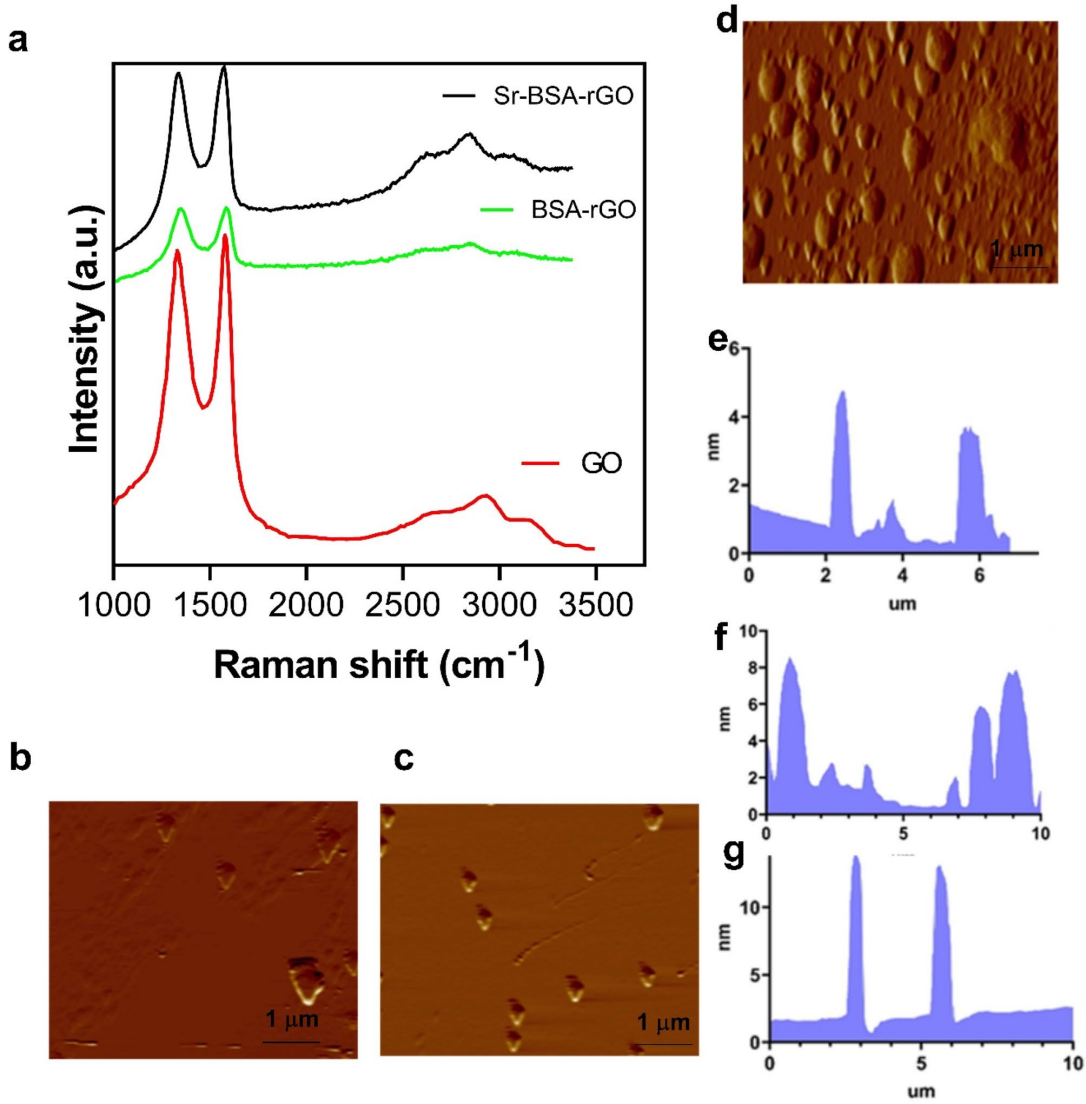
Structural and morphology of nanosheets. (**a**) Raman spectrum of GO, BSA-rGO and Sr-BSA-rGO nanosheets. (**b–d**) AFM images of GO, BSA-rGO, Sr-BSA-rGO nanosheets respectively. (**e–g**) The thickness profile of GO, BSA-rGO, and Sr-BSA-rGO respectively.

Raman spectroscopy is a helpful technique for characterizing carbon-based nanosheets, because they provide valuable information in the crystal structure of graphene-based materials. A variety of oxygen groups from oxidation of graphite, establish extremely irregular structure of GO. Figure 3a shows the Raman spectra of GO, BSA-rGO and Sr-BSA-rGO nanosheets. All patterns exhibited two major peaks corresponding to the D and G bands. The D band revealed the presence of carbon sites that were incomplete and disordered while the G band was associated with carbon atoms that were organized^29^. The D and G bands (I_D_/I_G_) were the critical parameters for determining ordered and disordered graphitic structure^27^. We understand the structural transforms because of the increase or decrease of the relative Intensity Ratio (I_D_/I_G_). The Raman spectrum of the BSA-rGO exhibited two peaks at 1347 cm^−1^ and 1599 cm^−1^ related to the D and G bands, respectively. The G band of BSA-rGO corresponds to the restoration of the defects according to the hexagonal network of carbon atoms^30^. The relative I_D_/I_G_ ratio was 1.108 that confirmed the GO structure reduction process, making a high amount of constructional defects^30^. After strontium adsorption, the D band stayed at 1347 cm^−1^, and the G band moved to 1594 cm^−1^. The slight growth of G band intensity resulted in a decrease of (I_D_/I_G_: 0.976) ratio, indicating a strong interaction between BSA-rGO hybrid and strontium. Metallic SrNPs improved the disordered structure of BSA-rGO which confirmed that there was a strong interaction with the successful crosslinking of strontium in defective sites of BSA-rGO^27^. The samples were prepared for AFM imaging by drop-casting on the glass slide and the solvent was evaporated at room temperature overnight. Figure 3b-g shows the various AFM images and height profile measurements. According to the height profile of AFM images, the thickness of BSA-rGO has surpassed in comparison with the thickness of GO, suggesting that BSA may have been adsorbed onto rGO as a stabilizer through hydrophobic and π-π stacking interactions. Another finding was that the typical average thickness of the Sr-BSA-rGO nanocomposite was more significant than the typical average thickness of RGO attributed to the presence of SrNPs on the surface. Increment in the thickness of graphene nanosheets after coating, has been previously reported. For example, Upadhyay et al. reported that the incensement in the thickness of GO nanosheets from 1 to 110 nm, was detected when the polyethylene was grafted into the surface of GO^31^. In the another work, reduction and decoration of GO nanosheets with polydopamine, enhanced the thickness of GO nanosheets^32^. Previously, we also utilized a monoclonal anti-body named Herceptin for in situ synthesizing and labelling graphene sheets, and we found enhancement of the graphene thickness by introducing anti-body^33^.

**Figure 4:**
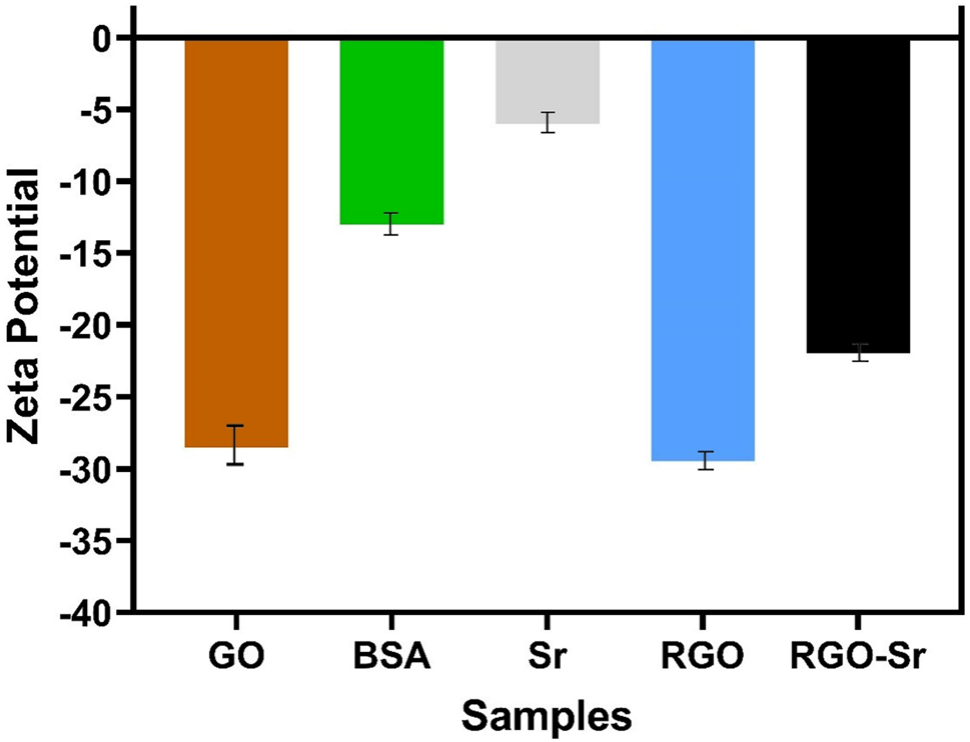
Zeta potential of GO, BSA, Sr, BSA-rGO (illustrated as RGO), and Sr-BSA-rGO (illustrated as RGO-Sr).

According to our findings, the zeta potential value for GO is −28.5 mV (Fig. 4). That is mainly due to oxygen functional groups on the surface of GO and residual negative charge densities. However, BSA-rGO voltage decreases to −29.47 mV that becomes more negative compared to GO. It shows that BSA-rGO had a more stable aqueous dispersion than GO, because of the BSA hydrophilic segments. SrNPs had a zeta potential value of −6 mV due to their low solubility in water, whereas the Sr-BSA-rGO represented a zeta potential value of −22.03 mV. The difference between BSA-rGO and Sr-BSA-rGO indicated the well attachment of metallic strontium on BSA-rGO. Despite this bond, the Sr-BSA-rGO nanosheets had a negative value, indicating stability in an aquatic environment.

**Figure 5:**
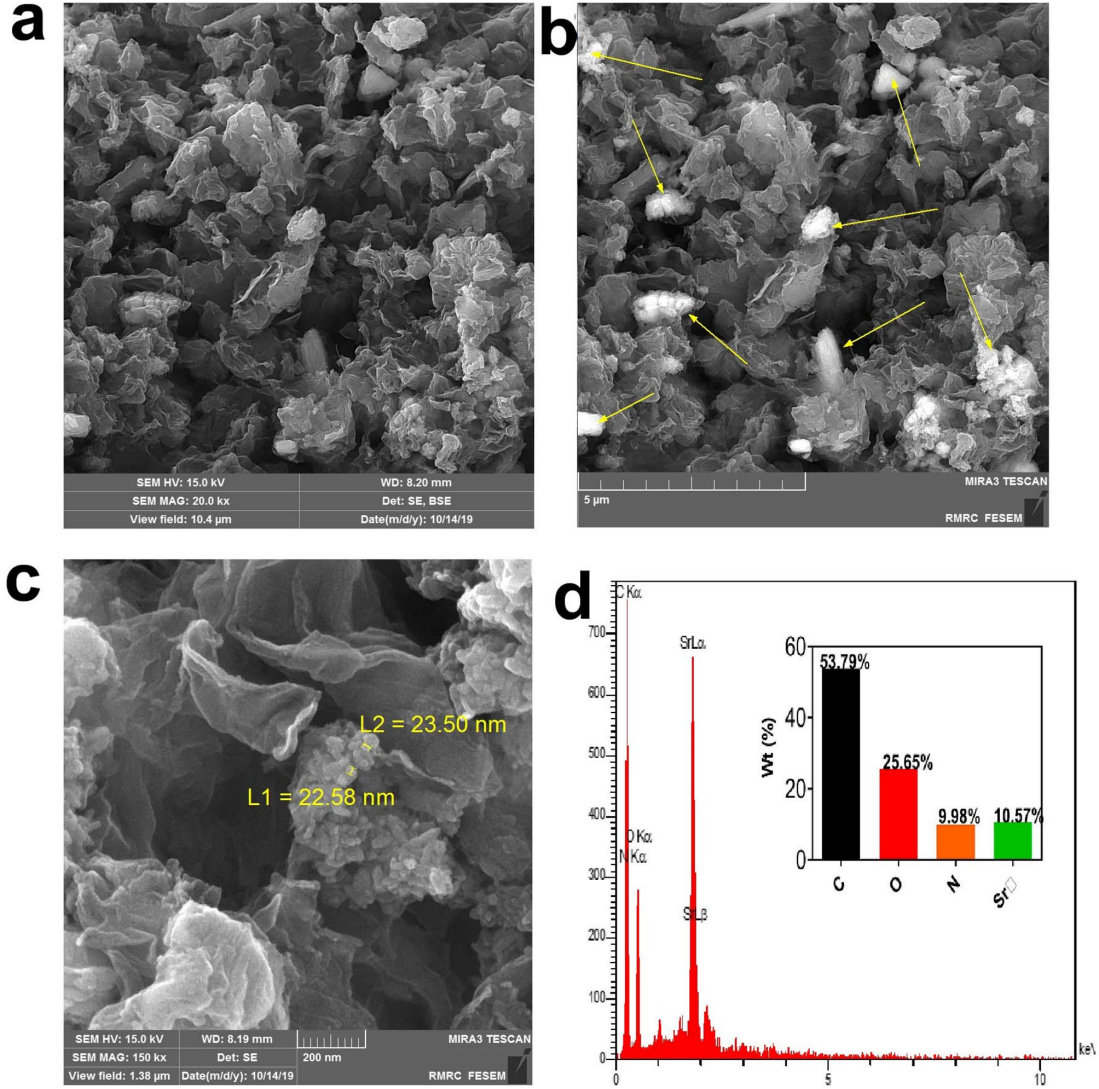
FESEM images of Sr-BSA-rGO nanosheets. (**a**) and (**c**) Secondary electron image. (**b**) Backscattered electron image (yellow arrows reveal Sr NPs). (**d**) EDS analysis of Sr-BSA-rGO nanosheets.

**Figure 6:**
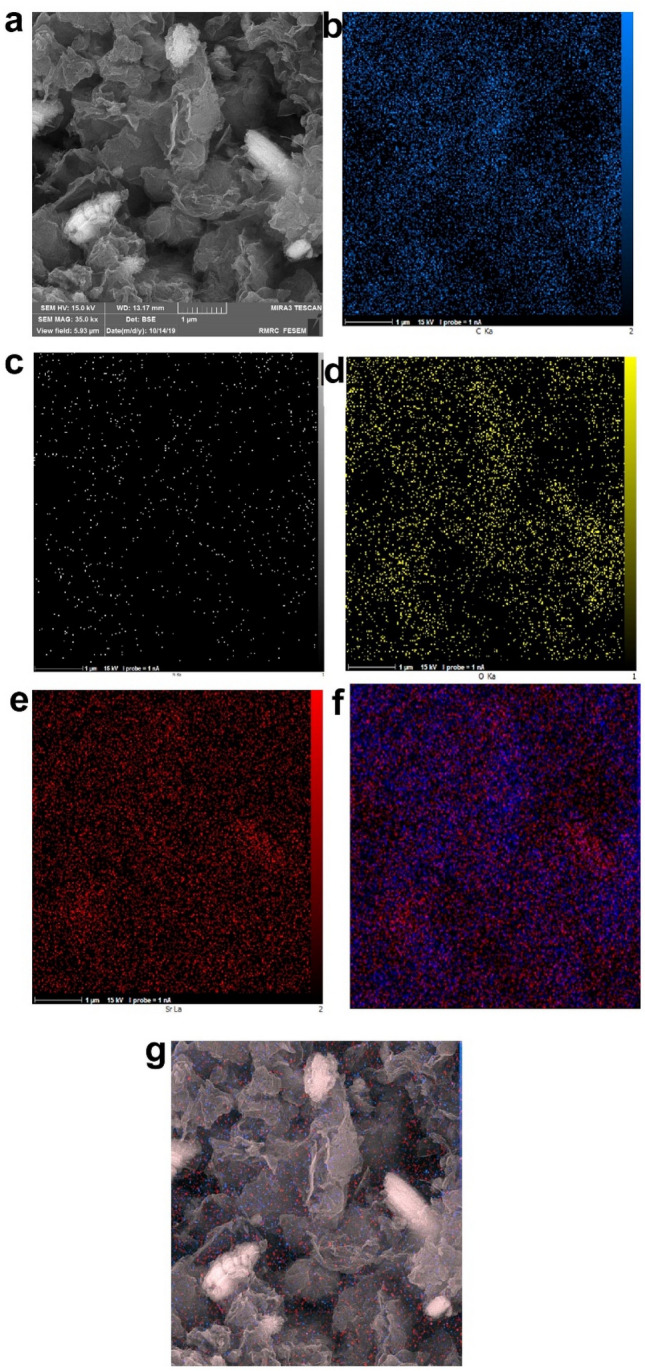
(**a–e**) Elemental mapping images of C, N, O and Sr elements of Sr-BSA-rGO. (**f**) Merged image of carbon and strontium. (**g**) Merged image of carbon and strontium in FESEM.

To confirm the assembling of SrNPs on the surface of BSA-rGO nanosheets, we used FESEM. As shown in the Fig. 5, SrNPs in dispersed and clustered form, were deposited on the surface of BSA-rGO nanosheets. We also used the backscattered electron option of FESEM for a better illustration of the SrNPs on the surface of the nanosheets (Fig. 5b). SrNPs have been observed lighter than graphene nanosheets. According to Fig. 5c, the size of SrNPs was observed near 20 nm and SrNPs possessed a spherical shape. The EDS spectrum of Sr-BSA-rGO exhibited that the appearance of the strontium with the weight percentage of 10.57%, was clear evidence for the effective decoration of SrNPs on the large surface area of BSA-rGO (Fig. 5d). The dispersion of SrNPs on the surface of BSA-rGO was monitored by element mappings analysis. The hybrid nanocomposite contained C, O, N and Sr as the main components, and each element was dispersed uniformly (Fig. 6), which was in accordance with FESEM images.

**Figure 7:**
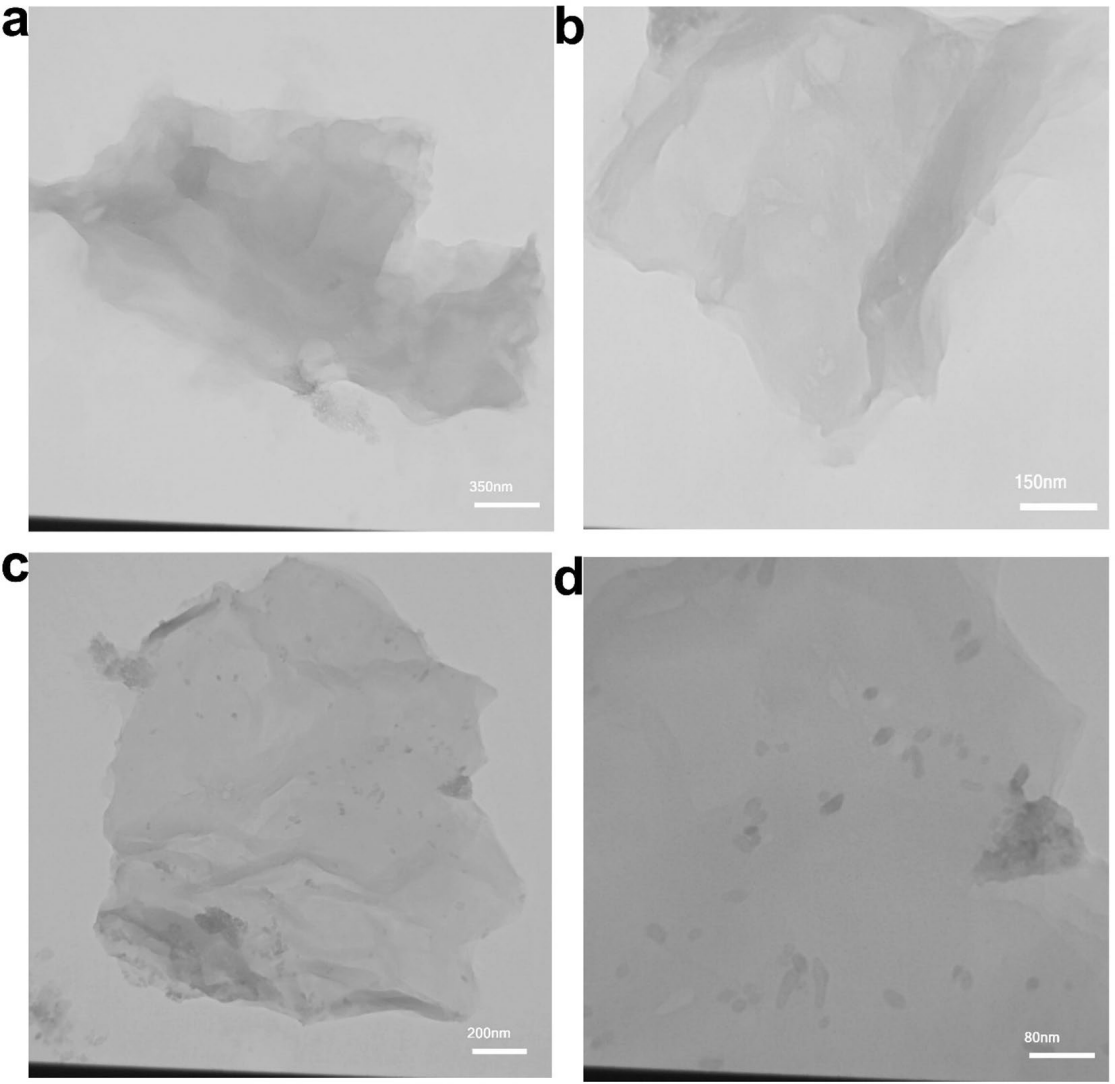
TEM images of (**a**) GO, (**b**) BSA-rGO and (**c**,**d**) Sr-BSA-rGO nanosheets. Black dots in the TEM images, were Sr nanoparticles.

Transmission electron microscopy (TEM) is an efficient technique for determining the grain size, size distribution and shape of the assembled NPs. Figure 7a,b illustrate TEM images of GO and BSA-rGO nanosheets^16^. There was no sign of aggregation in GO nanosheets after reduction with BSA, and the figure depicted the rough nanosheets that has been exfoliated already, revealing a folded wide surface area and clumped structure. These images supported our XRD and confirmed that there is no aggregation of GO nanosheets in presence of BSA. Figure 7c,d demonstrated well-dispersed strontium clusters and monodispersed NPs on the surface of BSA-rGO nanosheets). The black dots in Fig. 7c,d displayed the crystalline metallic strontium NPs that was identically dispersed on the surface of hydrophilic wrinkled rGO nanosheets.

**Figure 8:**
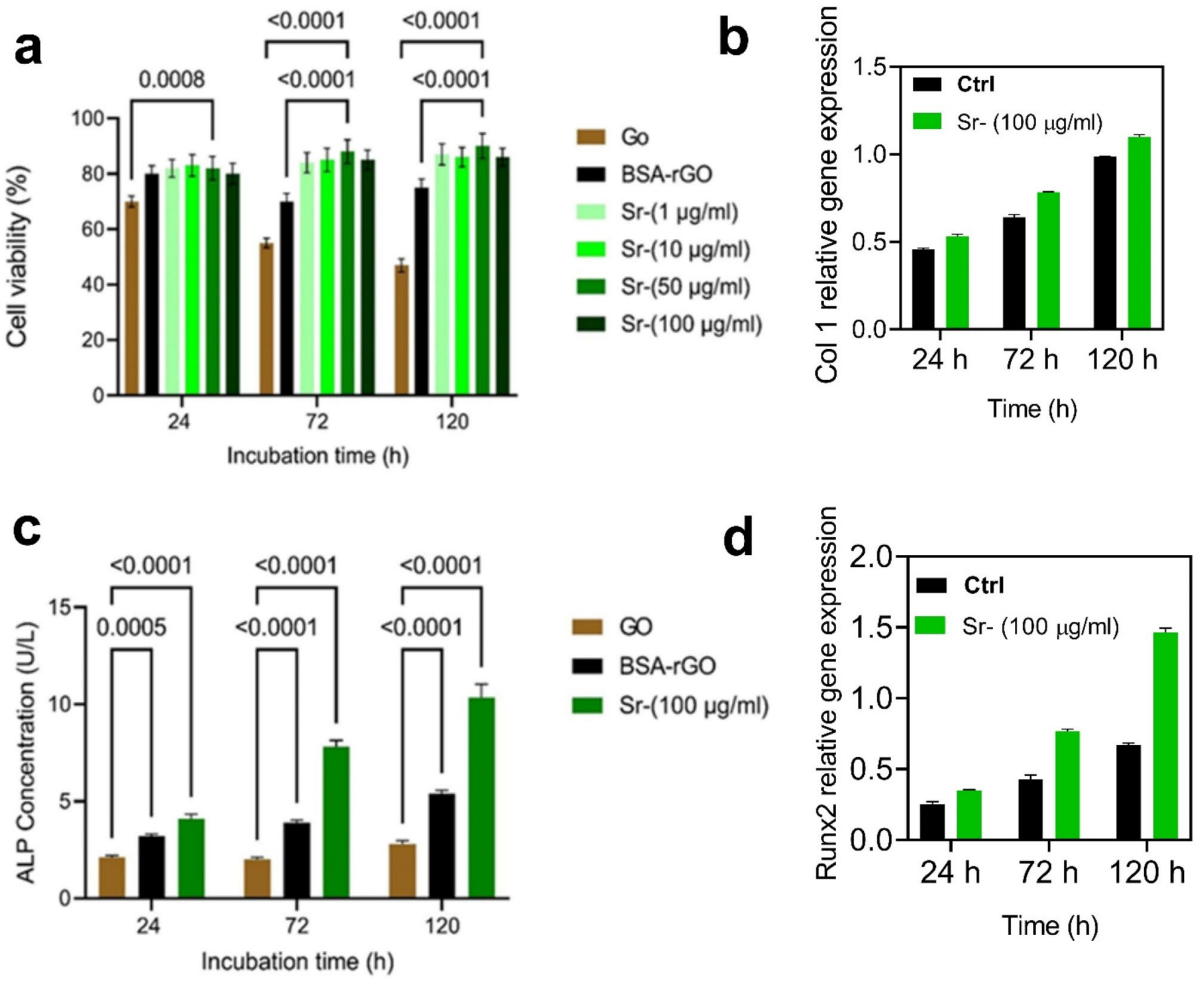
Biological assessment of the synthesized nanosheets. (**a**) Cell viability measurement (MTT assay) of MC3T3-E1 cells treated with different concentrations of the Sr-BSA-rGO (1, 10, 50, 100 μg/ml) and a constant concentration of GO and BSA-rGO (100 μg/ml) at various incubation times (24, 72 and 120 h). (**b**) ALP activity measurement of the MC3T3-E1 cells treated with a constant concentration of GO, BSA-rGO and Sr-BSA-rGO nanosheets (100 μg/ml) at different incubation times (24, 72 and 120 h). All experiments were conducted in triplicate (n = 3). (**c**) Effect of Sr-BSA-rGO on osteogenic differentiation of MC3T3-E1 cells. Sr-BSA-rGO nanosheets were used to treat the MC3T3-E1 cells in 100 g/ml. (**d**) Following an incubation period of 24, 72 and 120 h, RT-PCR was used to detect RUNX2 and Col 1A1 expression levels, and their normalization was done against GAPDH.

We studied the cytotoxicity of GO, BSA-rGO and Sr-BSA-rGO nanosheets using MTT assay. As shown in Fig. 8a, GO nanosheets decreased the viability of MC3T3-E1 cells to 70, 55 and 47% compared to control after incubation for 24, 72 and 120 h at a constant concentration (100 μg/ml), respectively. Cell viability percentages for 24, 72 and 120 h incubations with BSA-rGO were 80, 70 and 75%, respectively. BSA-rGO nanosheets increased the viability of cells compared to GO in the same concentration. For better studying the effect of Sr-BSA-rGO nanosheets on cells viability, we utilized various concentrations of Sr-BSA-rGO nanosheets (1, 10, 50, 100 μg/ml). The cell viability percentage was higher than 80% for cells incubated with different concentrations of Sr-BSA-rGO nanosheets. These nanosheets demonstrated the highest cell viability compared to GO and BSA-rGO. The toxicity of GO has been widely investigated that GO had negligible toxicity in high concentrations. In addition, protein-coated nanosheets and substrates had great potential for improving cell proliferation and viability. For example, Ahadian et al. reported an aqueous solution of graphene nanosheets by using BSA and graphite that were able to boost the cell viability and proliferation rate^22^. In addition, we found the enhanced cell viability for the groups treated with Sr-BSA-rGO nanosheets in different concentration. Due to the non-covalent interaction of SrNPs and graphene nanosheets, they can be released into the culture medium. Sr ions had capability in boosting the cell viability, especially in bone tissue engineering^15^.

Herein, we also studied the ALP activity of GO, BSA-rGO and Sr-BSA-rGO. The concentration of nanosheets was constant (100 µg/ml) and the experiment was followed for 24, 72 and 120 h. The Sr-BSA-rGO nanosheets boosted ALP activity of MC3T3-E1 cells compared to GO and BSA-rGO, that this trend was similar for 72 and 120 h (Fig. 8c). RT-PCR was utilized for assessing osteogenic differentiation of the MC3T3-E1cells-induced Sr-BSA-rGO. As shown in Fig. 8b,d, treatment with 100 μg/ml Sr-BSA-rGO significantly enhanced the expression of RUNX2 and Col1A1 genes. Interestingly, similar trend was observed in ALP activity and MTT assay. Following Sr-BSA-rGO treatment for 120 h, MC3T3-E1 cells exhibited highest level of ALP activity at the concentration of 100 μg/ml. Taken together, these experiments revealed that 100 μg/ml Sr-BSA-rGO elicited the most pronounced effects on the osteogenic differentiation of MC3T3-E1 cells.

## Conclusion

Briefly, we suggested a simple approach for in situ BSA-mediated synthesis of Sr-decorated rGO nanosheets according to the protein-based reduction/decoration. The UV–Vis, Raman, XRD and FTIR spectroscopy showed that BSA had a primary role in reducing and decorating SrNPs on the surface of rGO nanosheets. SEM and TEM images verified the deposition of SrNPs on the prominent surface area of BSA-rGO. After treatment with Sr-rGO nanosheets, MC3T3-E1 cells showed an enhanced ALP activity compared to GO and BSA-rGO, that this trend was observed in 72 and 120 h of treatment, respectively. In comparison to GO and BSA-rGO, cytotoxicity of Sr-BSA-rGO nanosheets in an MTT experiment, showed the best cell viability. After treatment of Sr-BSA-rGO for 120 h, MC3T3-E1 cells presented highest level of bioactivity at 100 μg/ml. On the whole, this study demonstrated that 100 μg/ml Sr-BSA-rGO established the most noticeable impacts on the osteogenesis and osteogenic differentiation of MC3T3-E1 cells. It seems that the use of synthetic hybrid nanobiomaterials may offer a viable route for the provision of bone substitute biomaterials in the context of tissue regeneration.

## Data Availability

The datasets used and/or analyzed during the current study are available from the corresponding author on reasonable request.
